# Influence of meteorological parameters in the seasonality of influenza viruses circulating in Northern Cameroon

**DOI:** 10.1111/irv.12612

**Published:** 2018-12-17

**Authors:** Hermann Landry Munshili Njifon, Chavely Gwladys Monamele, Cyprien Kengne Nde, Marie‐Astrid Vernet, Gake Bouba, Serges Tchatchouang, Mohamadou Ripa Njankouo, Raphaël Tapondjou, Louis Deweerdt, Wilfred Mbacham, Richard Njouom

**Affiliations:** ^1^ Centre Pasteur of Cameroon Yaounde Cameroon; ^2^ University of Yaoundé 1 Yaounde Cameroon; ^3^ National AIDS Control Committee Yaounde Cameroon

**Keywords:** influenza, meteorological parameters, Northern Cameroon, seasonality

## Abstract

**Background:**

Several studies have demonstrated the role of meteorological parameters in the seasonality of influenza viruses in tropical and subtropical regions, most importantly temperature, humidity, and rainfall.

**Objectives:**

This study aimed to describe the influence of meteorological parameters in the seasonality of influenza viruses in Northern Cameroon, a region characterized by high temperatures.

**Methods:**

This was a retrospective study performed in Garoua Cameroon from January 2014 to December 2016. Monthly proportions of confirmed influenza cases from six sentinel sites were considered as dependent variables, whereas monthly values of mean temperature, average relative humidity, and accumulated rainfall were considered as independent variables. A vector error correction model was used to determine the relationship between influenza activity and the meteorological variables.

**Results and conclusion:**

Analysis showed that there was a statistically significant association between overall influenza activity and influenza A activity with respect to average relative humidity. A unit increase in humidity within a given month leads to more than 85% rise in the overall influenza and influenza A activity 2 months later. Meanwhile, none of the three meteorological variables could explain influenza B activity. This observation is essential in filling the gap of knowledge and could help in the prevention and control strategies to strengthen influenza surveillance program in Cameroon.

## 1 INTRODUCTION

Influenza is a public health threat in the world as it is the source of important epidemics and pandemics.^1^ Several studies show that influenza is thought to infect approximately 1 billion people each year resulting in 3‐5 million severe cases and up to 500,000 deaths worldwide.^2^ In Africa, outbreaks of A/H3N2 influenza in Madagascar and the Democratic Republic of Congo in 2002 resulted in 754 and 170 deaths, respectively.^3^, ^4^ Influenza epidemiology is better known in temperate regions than in tropical regions.^5^, ^6^ In temperate regions, several studies have shown that temperature and relative humidity (RH) play an important role in the survival of the influenza virus^7^ and that influenza epidemics occur during the winter months. In tropical regions, there is a high level of influenza activity during the rainy season and epidemics occur from November to March.^8^, ^9^ However, it should be noted that influenza activity in tropical regions has a heterogeneous pattern without clear explanations.^2^, ^3^, ^4^, ^5^, ^6^

In these tropical settings, transmission of the disease seems to be continuous throughout the year; meanwhile, the exact period of occurrence of epidemics is difficult to predict.^10^, ^11^

In Cameroon, a sentinel surveillance network for influenza was implemented in 2007 by the Centre Pasteur of Cameroon and was later designated as the National Reference Centre for Influenza (NIC) for Cameroon by the World Health Organization (WHO). This public health activity concerned only the southern part of the country. The first results showed the circulation of A/H1N1/2009, A/H3N2, and B viruses^12^ with a high circulation in the rainy season.^13^, ^14^ A recent study performed in Yaounde found no significant association between influenza activity and three meteorological variables (temperature, rainfall, and RH) though some synchronies were observed with rainfall.^15^ The town of Garoua is located in northern Cameroon and is dominated by a Sudan tropical climate, with high temperatures that can exceed 30°C. In this city, the circulation of influenza viruses is poorly documented. Existing data are incomplete and does not provide information on the factors influencing the occurrence of the disease, with an inability to predict epidemics. Hence, this study was undertaken to explore the possible influence of meteorological variables on the circulation and transmission of influenza viruses in the North region of Cameroon. A retrospective analysis of the time series was carried out in the town of Garoua in the North region of Cameroon from January 2014 to December 2016, in order to evaluate a possible influence of the meteorological parameters in the seasonality of influenza.

## 2 METHODS

### 2.1 Study areas and design

**Figure 1:**
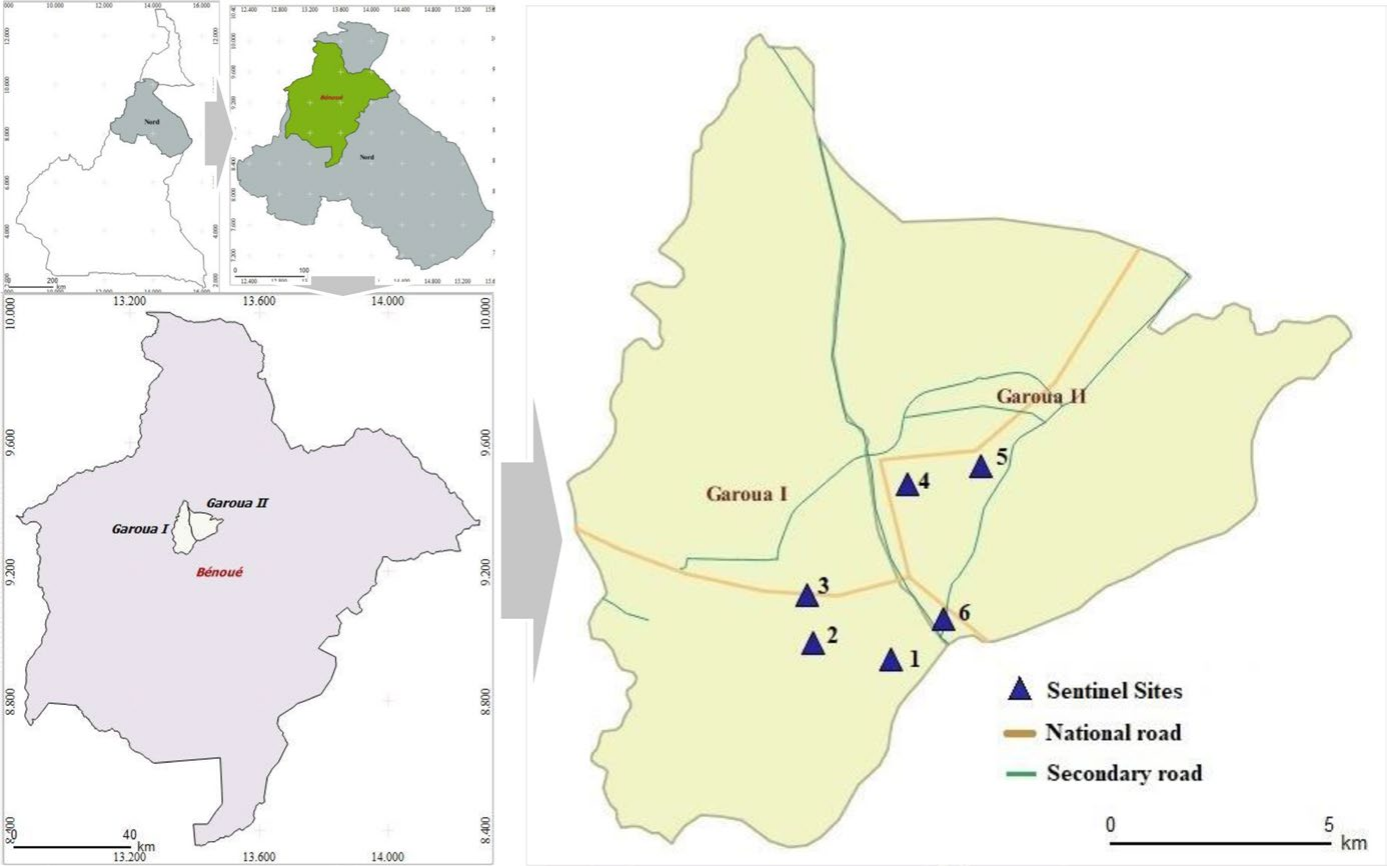
Map showing location of sentinel sites in Garoua. 1 = Souari Integrated Health Center (9.29°N, 13.39°E); 2 = Foulbéré Integrated Health Center (9.29°N, 13.37°E); 3 = Ouro‐Kanadji Integrated Health Center (9.30°N, 13.37°E); 4 = Roumdé‐Adja Integrated Health Center (9.32°N, 13.39°E); 5 = Poumpoumré Integrated Health Center (9.33°N, 13.41°E); 6 = Koota‐Lidjiré Integrated Health Center (9.30°N, 13.40°E)

Garoua is a port city and the capital of the North Region of Cameroon. It is located 9.30 latitude and 13.40 longitude and has a Sudan tropical climate with a dry season that runs from October to April and a shorter rainy season from May to September. The average annual rainfall amounts to 1000 mm. Temperatures remain high with an average of 28°C and maximum temperatures of 40‐45°C in March and April. However, large irregularities can be observed from one year to another and even from one month to another. Figure 1 shows the location of the study sites.

### 2.2 Epidemiological and virological data

Six health facilities of Garoua were involved in the influenza surveillance network in Cameroon. These health facilities consult individuals of all age groups. However, the majority of those with respiratory infections are children. During the study period, all participants were enrolled based on clinical definition for influenza‐like illness defined by a sudden onset of fever ≥38°C, cough, and/or sore throat with appearance in five previous days. All patients complying with this clinical definition were directly included in the study. After approving to participate in the study, socio‐demographic and clinical data were collected from each patient as well as nasal swabs. Swabs were introduced into a cryotube containing 2 mL of virus transport medium and stored at +4°C at study sites and were then transported to the Centre Pasteur of Cameroon where they were stored at −20°C prior to analysis and −80°C thereafter. In the laboratory, viral RNA was extracted from 140 μL of the sample using the QIamp viral RNA kit (Qiagen^®^ , Hilden, Germany) according to the manufacturer's recommendations. The RNA was eluted with 60 μL of RNase/DNase elution buffer provided by the manufacturer in the kit. Subsequently, the obtained RNA was subjected to the commercial RT‐PCR multiplex technique in an ABI Prism 7500 thermal cycler (Applied Biosystem, Foster City, CA, USA).

### 2.3 Meteorological data

The climate variables from January 2014 to December 2016 available for this study at the Meteorological Service of the Ministry of Transport of Cameroon were monthly cumulative rainfall, average monthly RH, and average monthly ambient temperature. These data were obtained from a ground station located at latitude 9°20′, longitude 13°23′E, and 242M altitude.

### 2.4 Ethical considerations

This study was approved by the National Ethics Committee and the Ministry of Public Health of Cameroon. All participants enrolled gave their signed informed consent for those over 21 years old or the legal representative (parents, tutors, or guardians) for those under 21. Participant data were kept confidential.

### 2.5 Statistical analysis

In order to study the temporal distribution of influenza per year, we considered the monthly proportion of respiratory samples that tested positive for influenza (number of respiratory samples that were tested positive for influenza virus divided by the number of samples tested during the month) to represent influenza activity. Data were analyzed using eviews software version 9. A VECM (vector error correction model) was used to estimate the model which best explains the variations in influenza activity. The following steps were followed in performing data analysis: study of the stationarity of the series, lag selection for co‐integration test and estimation of VECM, Johansen co‐integration test, VECM estimation which adjusts to both short run changes in variables and deviations from equilibrium, stability, and residual diagnosis. Stationarity of the series was verified using graphs, auto‐correlation function (ACF), and the augmented Dickey‐Fuller test (ADF). Meanwhile, residual diagnosis involved performing serial correlation LM test, heteroscedasticity test, and normality test. The threshold value for statistical significance was set at 0.05.

## 3 RESULTS

Throughout the study period, males and females represented 48.6% (405/833) and 51.4% (428/833) of enrolled patients, respectively. The 0‐5 years age‐group was the most represented. Most samples were collected in the beginning of the year between January and March. Out of the 833 samples, 23.3% (194/833) were positive for influenza virus (Table 1).

**Table 1:** Demographic characteristics of study population

	Overall (%)	2014 (%)	2015 (%)	2016 (%)
Gender				
Male	405 (48.6)	125 (49.4)	176 (48.8)	104 (47.5)
Female	428 (51.4)	128 (50.6)	185 (51.2)	115 (52.5)
Age‐group (y)				
0‐5	598 (71.8)	188 (74.3)	256 (70.9)	154 (70.3)
6‐20	130 (15.6)	35 (13.8)	57 (15.8)	38 (17.4)
21‐50	90 (10.8)	29 (11.5)	41 (11.4)	20 (9.1)
>51	15 (1.8)	1 (0.4)	7 (1.9)	7 (3.2)
Period				
January‐March	261 (31.3)	24 (9.5)	148 (41)	89 (40.6)
April‐June	259 (31.1)	99 (39.1)	120 (33.2)	40 (18.3)
July‐September	153 (18.4)	36 (14.2)	68 (18.8)	49 (22.4)
October‐December	160 (19.2)	94 (37.2)	25 (6.9)	41 (18.7)
Influenza results				
Positive	194 (23.3)	56 (22.1)	91 (25.2)	47 (21.5)
Negative	639 (76.7)	197 (77.9)	270 (74.8)	172 (78.5)
Total	833	253	361	219

Based on monthly data, there were 36 observations from January 2014 through December 2016. Table 2 summarizes the descriptive statistics of each variable. With respect to meteorological variables, the North region had relatively high temperature ranging from 25.1 to 34.6°C (mean: 28.8) with average precipitations of approximately 60 mm/month and high variations in humidity between 25% and 80%.

**Table 2:** Characteristics of study variables

	Min	Max	Mean	SD	Median	IQR
Meteorological variables (monthly)						
Mean temperature (°C)	25.1	34.6	28.8	2.6	28.3	26.9‐30.3
Accumulated rainfall (mm)	0	316.4	60.7	82.1	24.2	0‐89.6
Average relative humidity (%)	25	80	55	20	56	35‐75
Laboratory data (monthly counts)						
Samples collected	0	82	24.5	20.5	19	13‐28
Overall influenza cases	0	29	5.4	6.8	3	0‐9
Influenza A	0	16	3.4	4.6	1	0‐5.5
Influenza B	0	16	1.9	3.7	0	0‐1.5

Min, minimum; Max, maximum; SD, standard deviation; IQR, inter quartile range.

**Figure 2:**
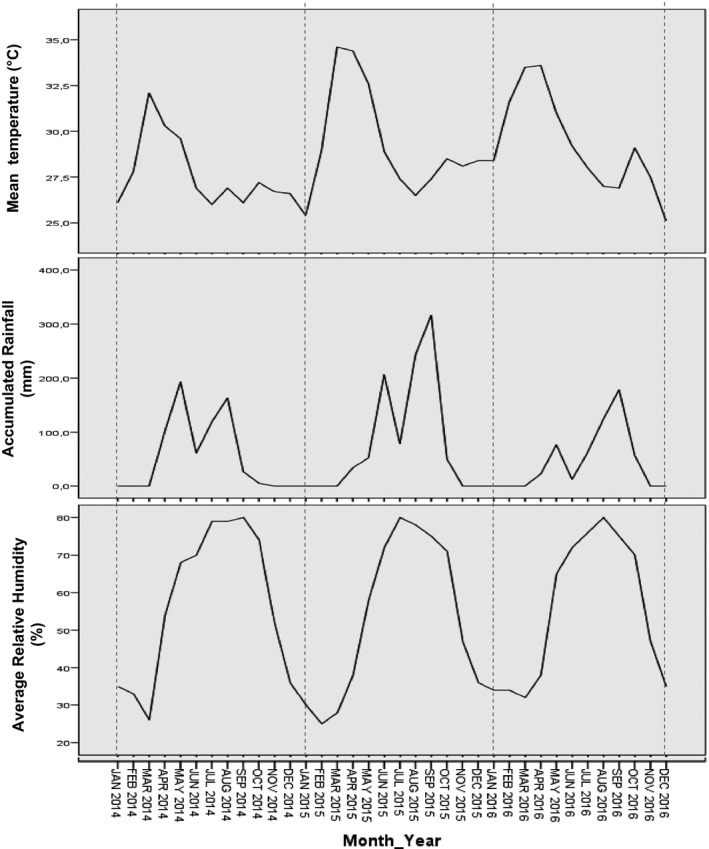
Monthly distribution of meteorological variables

**Figure 3:**
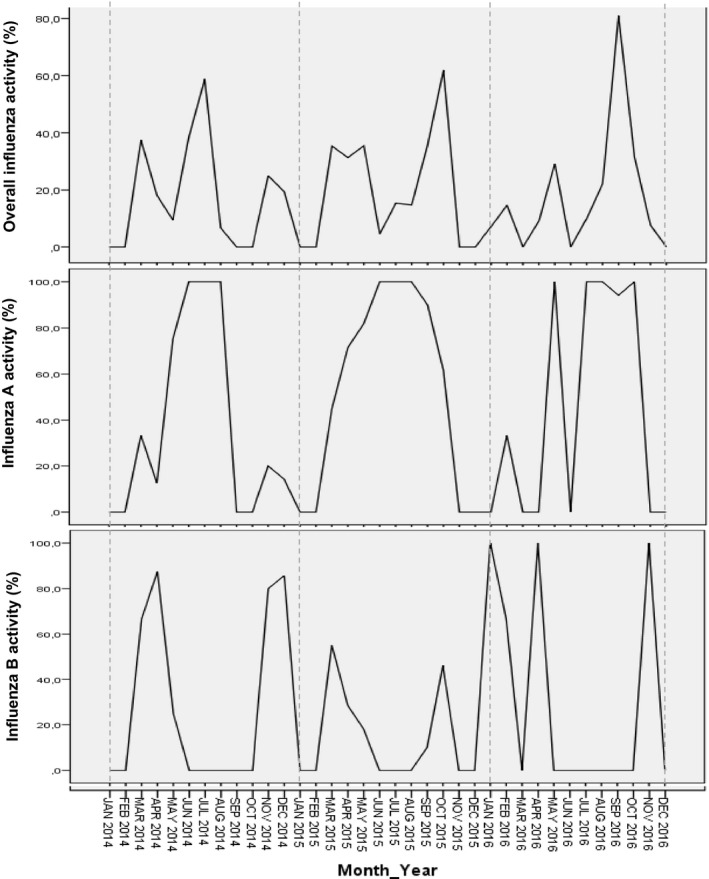
Monthly distribution of influenza activity

Figures 2 and 3 show the trends observed between meteorological variables and influenza activity. Two major peaks were observed in the circulation of overall influenza cases in the Northern region of Cameroon. We noted no temporal relationship between the three weather variables. Influenza A and B subtypes however showed some synchronies with two meteorological variables. Influenza A virus was positively correlated to rainfall while influenza B virus was inversely correlated to RH.

As previously stated in the methods section, the three models were evaluated for the respect of conditions necessary for VECM analysis. All variables were found to be stationary when differentiated (*I*(1)) based on the results of the ACF and the ADF test (*P*‐value < 0.05). The optimal lag was determined using the VAR (vector autoregression) lag order selection criteria and was found to be lag 2 since it is the one which best minimized the different information criterion. The Johansen co‐integration test identifies stable, short‐run and long‐run relationships between sets of variables. It revealed the existence of four co‐integration relationships between the different influenza types and the three meteorological variables (Table 3). This means that four linear combinations exist between the variables that causes them to have a short‐term and/or long‐term relationship over time. Stability of the model was verified using the CUMSUM recursive estimates, and the models were found to be stable. All residuals were not autocorrelated, had no problem of heteroscedasticity, and were found to be white noises.

**Table 3:** Results of the Johansen co‐integration test

Model	Number of CE	Eigenvalue	Trace statistic	*P*‐value
Overall influenza activity	None	0.5852	47.8561	<0.0001
	At most 1	0.4625	54.1652	<0.0001
	At most 2	0.4124	33.0564	0.0001
	At most 3	0.3563	14.9771	0.0001
Influenza A activity	None	0.5852	47.8561	<0.0001
	At most 1	0.5639	80.6105	<0.0001
	At most 2	0.4568	52.3872	0.0001
	At most 3	0.3386	31.6359	0.0002
Influenza B activity	None	0.6632	90.1062	<0.0001
	At most 1	0.4547	53.1045	<0.0001
	At most 2	0.3971	32.4890	0.0001
	At most 3	0.3621	15.2838	0.0001

CE, Co‐integration equations.

The estimation of the different models is given in Table 4. The model for overall influenza activity was the best model since it could explain up to 65.2% of the variations in influenza activity followed by that for influenza A activity (46.2%) and influenza B activity (42.9%). It is noted that only one meteorological variable best explains the variation of overall influenza and influenza A activity, that is, RH at lag 2. A unit increase in humidity within a given month leads to more than 85% rise in the overall influenza and influenza A activity two months later. Overall influenza activity was also found to be influenced by its preceding activity. Indeed, a unit rise in overall influenza activity during a given month leads to a 49.8% rise in this same variable during the following month.

**Table 4:** Estimates of VEC model with significant threshold

Models	Optimal lag	*R* ^2^ (%)	Variable	Lag	Coefficient	*P*‐value
Overall influenza activity	2	65.2	Overall influenza activity	1	0.4980	0.0431
			Average relative humidity	2	0.8779	0.0269
Influenza A activity	2	46.2	Average relative humidity	2	0.9767	0.0433
Influenza B activity	2	42.9	None	NA	NA	NA

NA, not applicable.

## 4 DISCUSSION

In this study, we evaluated the association between influenza activity and meteorological variables in six sentinel sites of influenza surveillance in the Northern region of Cameroon (Garoua) which is characterized by a Sudan tropical climate. Data from 2014 to 2016 show that there are two major peaks in the circulation of influenza virus. These results are different from those found in the Centre region of Cameroon by Heraud et al. (2008‐2009),^16^ Kenmoe et al,^14^ and Monamele et al^15^ who found that influenza circulated throughout the year or with a seasonal peak (May through November) . On the other hand, our results corroborate with reports of Caini et al^18^ who observed a heterogeneous pattern in the circulation of influenza virus across several tropical countries since none of the peaks observed in this study were overlapping.

Average RH was the only meteorological variable to show a significant and positive association to overall influenza activity and to influenza A activity. Several studies have instead reported a negative association between influenza and RH.^19^ This is due to the fact that respiratory particles exposed to low ambient RH between 20% and 35% can remain airborne for up to 24 hours, thus increasing the risk of airborne transmission.^19^, ^20^ More studies are required to understand the reason for the rise in overall influenza activity and influenza A activity despite high RH in the North region. Previous reports from tropical regions particularly in Bangladesh, Guatemala, El Salvador, and Panama also revealed a proportional association between influenza and humidity [^2,9,20^]. Since airborne transmission is unlikely in these circumstances, transmission of influenza probably occurs through contact and droplet modes.^24^ On the other hand, none of the variables could explain influenza B activity. A recent report by Yang et al has shown that association of influenza virus with weather variables differed with respect to sub‐types.^22^

Accumulated rainfall and mean temperature were not associated with influenza activity in the North region. Previous studies have shown varying relationships between rainfall and influenza activity with respect to the region. Studies from Ivory Coast,^23^ Thailand,^24^ Honduras, and Nicaragua^25^ have shown a significant correlation with the number of influenza cases. Meanwhile, a negative correlation between the number of influenza cases and rainfall has been reported by Murray et al^25^ in cases of influenza A (H5N1). Increase in influenza transmission during rainfall can be explained by an increase in indoor crowding which favors aerosol and contact transmission.^23^

With regard to temperature, the North region is characterized by relatively high temperatures ranging from 25.1 to 34.6°C as opposed to the Centre region with temperatures varying between 21.3 and 27.6°C.^15^ This could in part explain the relatively low number of cases of influenza virus in the North region as compared to the Centre region since transmission of this virus is less efficient at high temperatures.^15^ We noted no statistically significant association between temperature and influenza activity. Interestingly, and unlike previous reports which showed that influenza transmission is abolished at temperature ≥30°C,^19^ we noted influenza activity at these temperatures. Animal studies performed in laboratory controlled settings have shown that increasing ambient temperature during transmission experiments prevents airborne transmission but not contact transmission. Showing that transmission of influenza in Garoua probably occurs by the contact mode.

A major limit to this study was the relatively short period from which data were obtained as well as the use of monthly data instead of weekly data. Moreover, there were many months for which influenza activity was null due to low or limited samples collected. All these might have affected the precision and the power of our statistical analyses. More data are required from this region in order to confirm the trends observed in this study and to obtain more conclusive results. Also, it will be essential to consider other environmental factors not included in this study such as UV radiation, absolute humidity, air quality, and wind which have been reported to influence influenza transmission as well in order to rule out the possibility of spurious relationships.

This study enabled to fill the gap of knowledge on the circulation of influenza virus in the North region of Cameroon which has a characteristic Sudan tropical climate. Among the three meteorological variables considered in this study, influenza activity in Garoua could only be explained by variations in average RH. This observation could help in the prevention and control strategies to strengthen the influenza surveillance program in Cameroon.

## COMPETING INTERESTS

The authors declare that they have no competing interests.
